# Lehrprojekt zu Priorisierungskriterien in der medizinischen Versorgung

**DOI:** 10.1007/s00481-022-00690-1

**Published:** 2022-04-04

**Authors:** Magdalène Lévy-Tödter

**Affiliations:** Hochschulzentrum Hamburg, FOM Hochschule, Schäferkampsallee 16a, 20357 Hamburg, Deutschland

## Ziele und Aufbau des Lehrprojekts

Seit Längerem werden der Umgang mit knappen Ressourcen und bevorzugte Strategien wie Effizienzsteigerung (Rationalisierung) oder Selbstbegrenzung (Rationierung und Priorisierung) im Gesundheitswesen diskutiert (u. a. Marckmann und Siebert 2002; ZEKO 2007; Du Bois und Dörr 2014). Aus didaktischer Perspektive bietet eine Auseinandersetzung mit der Priorisierung im Sinne einer „ausdrücklichen Festlegung von Vor- und Nachrangigkeiten für die Behandlung bestimmter Krankheiten“ (Rosenberger 2019, S. 99) die Möglichkeit, in Lehrveranstaltungen die Bedeutung formaler und inhaltlicher Kriterien für eine gerechte Verteilung begrenzter Güter im Gesundheitswesen zu thematisieren. Krisen wie die Covid-19-Pandemie haben gezeigt, wie wichtig es ist, Gesundheitsfachkräfte nicht nur über Priorisierungsregeln für Triage-Situationen zu informieren, sondern sie in die Lage zu versetzen, in einem Priorisierungsprozess medizinische und außermedizinische Kriterien anzuwenden und ggf. kritisch zu hinterfragen (Stoecker 2020).

Im Modul „Ethik im Gesundheitswesen“ an der FOM Hochschule für Oekonomie und Management gGmbH sollen Studierende des berufsbegleitenden Bachelor-Studiengangs „Gesundheits- und Sozialmanagement“ sich unter anderem mit den ethischen Dimensionen von Rationalisierungs‑, Rationierungs- oder Priorisierungsmaßnahmen im Gesundheits- und Sozialwesen auseinandersetzen. Die hohe Heterogenität der Studierendenschaft hinsichtlich der Bildungsbiografien oder Berufserfahrungen bietet die Chance, die Vielfalt der gesellschaftlichen Wertvorstellungen unter den Studierenden für die Lehre zu Fragen der Gestaltung von Beteiligungsprozessen fruchtbar zu machen. Das Lehrprojekt im Fokus dieses Beitrags nimmt eine mittlere Position zwischen einem Planspiel (häufig mit hohem Organisationsaufwand verbunden) und einem Rollenspiel (Geuting 1992) ein. Es soll den Studierenden in der Simulation einer Arbeitssitzung einer hypothetischen Ethikkommission für Priorisierungsfragen die Möglichkeit geben, das erworbene Wissen über die Priorisierung in der medizinischen Versorgung in konkretes Handeln zu überführen. Ziel ist es, ihnen in einer dreistündigen Sitzung ein Grundverständnis für die Entwicklung von gerechten Priorisierungskriterien in einem Partizipationsverfahren zu vermitteln. Das Lehrprojekt besteht aus drei aufeinander bezogenen Phasen.

### Einführungsphase

In einem ersten Schritt werden in einem Kurzvortrag ethische Aspekte der Priorisierung in der medizinischen Versorgung präsentiert. Dazu gehören die Definition und Abgrenzung zentraler Begriffe wie Rationalisierung, Rationierung und Priorisierung (Marckmann 2010; Marckmann und in der Schmitten 2015). Nach einer kurzen Vorstellung des sogenannten „Oregon Health Plan“ (USA), im Rahmen dessen eine Priorisierungssetzung (mit Prioritätsgrad und -rang) von mehreren Hunderten von Leistungen des Gesundheitswesens unter Einbezug der Bevölkerung des US-Bundesstaates Oregon erstellt wurde (Marckmann und Siebert 2002), erfolgt ein Einstieg in die wissenschaftliche Diskussion im deutschsprachigen Raum über eine transparente und gerechtigkeitsorientierte Setzung von Prioritäten in der medizinischen Versorgung anhand von Auszügen mehrerer Stellungnahmen (ZEKO 2007; Du Bois und Dörr 2014). Aufgrund seiner Aktualität bietet der Kommentar von Ralf Stoecker zur Verteilung knapper Ressourcen in der Intensiv- und Notfallmedizin während der Corona-Pandemie (Stoecker 2020) eine gute Ergänzung zu den oben geführten Inputs. Die Spezifizierung von formalen und inhaltlichen Kriterien wie Konsistenz, Legitimität, medizinische Bedürftigkeit, erwarteter medizinischer Nutzen und Kosteneffektivität (ZEKO 2007; Du Bois und Dörr 2014; u. a.) ist ein wichtiger Arbeitsschritt in der ethischen Bewertung von Public Health Maßnahmen. Die Darstellung der Entwicklung eines Katalogs ethischer Bewertungskriterien in Form einer Narration mit konkreten Etappen („Oregon Health Plan“; ZEKO 2007; u. a.) und nationalen und internationalen Akteuren (u. a. Deutsche Ärztekammer, Ethiker) soll den Studierenden nahebringen, dass sie ebenfalls Teil dieser Geschichte sein können, wenn sie durch einen differenzierten Umgang mit moralischen Wertekonflikten (Moritz 2017) zur Weiterentwicklung einer Public Health Maßnahme beitragen können.

### Simulationsphase

Zur Vorbereitung der Simulation bilden die Studierenden Kleingruppen. Es folgt eine Vorstellung des Projekts „Einstellungen zu Priorisierungen in der medizinischen Versorgung“ von der DFG-geförderten Forschergruppe FOR655 (Jacobs University Bremen). Das Projekt umfasst Interviews, Fokusgruppen sowie Befragungen von diversen Stakeholdergruppen (u. a. Deutscher Ärztinnenbund, Vertreter der Krankenkassen) sowie eine repräsentative Bevölkerungsbefragung und erstreckte sich über den Zeitraum von 2009 bis 2015 (Diederich und Schreier 2010; Diederich et al. 2015). Die Studierenden sollen sich nun vorstellen, dass sie als Mitglieder einer hypothetischen Ethikkommission für Priorisierungsfragen beauftragt worden sind, aus den Antworten von Stakeholdergruppen- und Bevölkerungsbefragungen inhaltliche Priorisierungskriterien zu identifizieren und daraus Handlungsempfehlungen für die Politik abzuleiten. Jede Gruppe erhält Ausschnitte der Studienergebnisse in Tabellenform ohne Interpretation. Zur Begründung des partizipativen Ansatzes in diesem umfassenden Forschungsprojekt eignet sich das Positionspapier des Ethikausschusses des Deutschen Ärztinnenbundes zur Priorisierung medizinischer Leistungen, in dem erläutert wird, warum „faire Partizipationsmöglichkeiten“ dazu führen können, mit potenziellen „Interessenkonflikten verschiedener Gruppen – Patientenvertreter, Ärzteschaft, Kostenträger, Pharmaindustrie“ (Du Bois und Dörr 2014, o. S.) transparent umzugehen.

Die Dokumente sind bereits nach Themenbereichen untergliedert. In der eigentlichen Spielphase soll jede(r) Studierende je nach Länge der Tabellen ein oder zwei Themenbereiche (z. B. Gesundheitsverhalten als Priorisierungskriterium) bearbeiten. Anhand einer Frage zu verhaltensbezogenen Priorisierungskriterien (Diederich und Schreier 2010, S. 23) soll exemplarisch aufgezeigt werden, wie die Fragebögen von FOR655 aufgebaut sind. Auf die Frage „Es gibt verschiedene gesundheitsgefährdende Verhaltensweisen, die das allgemeine Krankheitsrisiko erhöhen können. Bei welchen der folgenden gesundheitsgefährdenden Verhaltensweisen sollte Ihrer Meinung nach der Patient höhere Zuzahlungen leisten?“ werden gesundheitsgefährdende Verhaltensweisen wie „ungesunde Ernährung“, „hoher Alkoholkonsum“, „Rauchen“, „Extremsport (z. B. freies Klettern, Klippenspringen)“ oder ähnlich „Bewegungsmangel“ aufgelistet. Die Antwortmöglichkeiten lauten in diesem Fall „Ja“, „Nein“, „Weiß nicht“ und „Antwort verweigert“. Im Anschluss daran haben die Studierenden 60 min, um eine kurze Präsentation ihrer Analyse und Empfehlungen vorzubereiten. Als Unterstützung erhalten sie folgende Fragen: Welche Allokationspräferenzen in der deutschen Bevölkerung und Ärzteschaft lassen sich anhand der Antworten identifizieren? Welche fünf Handlungsempfehlungen können Sie daraus ableiten? Im Anschluss an die Kurzpräsentationen findet eine Plenumsdiskussion über die konkret erfolgte Ableitung von Handlungsempfehlungen für die Allokation und Finanzierung medizinischer Leistungen statt. Die Studierenden werden unter anderem darum gebeten, ihr Verständnis der Kriterien mit dem der Forschung zu vergleichen. Die Simulationsphase zielt darauf ab, die Partizipationsbereitschaft der Studierenden an der Entwicklung von Maßnahmen in der medizinischen Versorgung zu fördern, indem sie an „realitätsnahen Probehandlungen“ (Engartner et al. 2015, S. 189) teilnehmen können.

### Reflexionsphase bzw. De-Briefing

Die Sitzung endet mit einer Abschlussreflexion über die ethischen Aspekte von Priorisierung im Gesundheitswesen und über die Bedeutung einer öffentlichen Diskussion über das Thema. Wie die Beschreibung erkennen lässt, besteht in diesem Lehrprojekt ein fließender Übergang zwischen der Spiel- und Reflexionsphase. Die Selbstreflexion wird gefördert, indem einzelne Studierende nach ihrer persönlichen Lernerfahrungen gefragt werden. Diese Phase dient dazu, den Studierenden die Möglichkeit zu geben, sich über ihre Einschätzung des Transfers des Gelernten für die berufliche Praxis auszutauschen. Gerade bei umstrittenen Themen wie der Priorisierung ist es wichtig, über Partizipationspotenziale der Bevölkerung und der eigenen Person zu diskutieren (Engartner et al. 2015).

## Reflexionen über das Lehrprojekt

Die Interaktion zwischen der Lehrkraft und den Studierenden variiert demnach von Phase zu Phase. Während der Einführungsphase stellt die Lehrkraft in Form eines Vortrags die Hintergründe des Handelns dar. In der Simulationsphase hat die Lehrkraft eher einen Beobachterstatus. In der Reflexionsphase dient ihre Moderation vor allem dazu, die Begründungsmuster der Studierenden herauszuarbeiten. Aufgrund der hohen Heterogenität der Studierenden im Hinblick auf Alter, beruflichen Hintergrund und eigene Wertvorstellungen führt die Besprechung der Ergebnisse und Ableitung von Handlungsempfehlungen häufig zu lebhaften Diskussionen. Dies gilt insbesondere für Priorisierungskriterien wie gesundheitsbezogenem Risikoverhalten, da die Posteriorisierung bei Verletzungen aufgrund von Risikosportarten (Diederich 2011) einerseits mit der Frage der Eigenverantwortung gekoppelt ist. Andererseits zeigen die Diskussionen in der Studierendengruppe, dass die Bestimmung von „Risikoverhalten“ eine Generationenfrage zu sein scheint. Die Herausforderung in dieser Phase ist das Zurückführen der Diskussion auf eine sachliche Ebene. Dieses Lehrprojekt ist bisher nicht evaluiert worden.

## References

[CR3] Diederich A, Diederich A, Koch C, Kray R, Sibbel R (2011). Einstellungen zu Priorisierungen in der medizinischen Versorgung. Ergebnisse einer repräsentativen Bevölkerungsbefragung. Priorisierte Medizin. Ausweg oder Sackgasse der Gesundheitsgesellschaft?.

[CR2] Diederich A, Schreier M (2010). Einstellungen zu Priorisierungen in der medizinischen Versorgung: Ergebnisse einer repräsentativen Bevölkerungsbefragung. Priorisierung in der Medizin, FOR 655 Nr. 27/2010.

[CR1] Diederich A, du Bois G, Dörr D (2015). Einstellungen zu Priorisierungen in der medizinischen Versorgung: Ergebnisse einer Befragung des Deutschen Ärztinnenbundes (DÄB). Priorisierung in der Medizin, FOR 655 Nr. 40/2015.

[CR4] Du Bois G, Dörr D (2014) Positionspapier des Ethikausschusses des Deutschen Ärztinnenbundes zur Priorisierung medizinischer Leistungen. Deutscher Ärztinnenbund e. V. https://www.aerztinnenbund.de/Positionspapier_des_Ethikausschusses_des.2225.0.2.html. Zugegriffen: 21. Okt. 2021

[CR5] Engartner T, Siewert MB, Meßner MT, Borchert C (2015). Politische Partizipation „spielend“ fördern? Charakteristika von Planspielen als didaktisch-methodische Arrangements handlungsorientierten Lernens. Z Politikwiss.

[CR6] Geuting M (1992). Planspiel und soziale Simulation im Bildungsbereich.

[CR7] Marckmann G (2010). Ethische Grundlagen der Priorisierung im Gesundheitswesen. Bundesgesundheitsblatt.

[CR9] Marckmann G, in der Schmitten J, Marckmann G (2015). Medizinische Entscheidungen unter Knappheitsbedingungen. Praxisbuch Ethik in der Medizin.

[CR8] Marckmann G, Siebert U (2002). Kosteneffektivität als Allokationskriterium in der Gesundheitsversorgung. Z Med Ethik.

[CR10] Moritz A, Eichler U, Moritz A (2017). Ein Kompetenzentwicklungsmodell für den Ethikunterricht. Ethik kompetenzorientiert unterrichten I und II. Eine Konzeption für die Klasse 9/10. Begleitmaterial.

[CR11] Rosenberger M, Fuchs M, Greiling D, Rosenberger M (2019). (Wie) Kann Rationierung fair sein? Ethische Überlegungen zur Ressourcenbegrenzung im Gesundheitswesen. Gut versorgt? Ökonomie und Ethik im Gesundheits- und Pflegebereich.

[CR12] Stoecker R (2020) Verteilung knapper Ressourcen in der Intensiv- und Notfallmedizin. Ein ethischer Hintergrundkommentar zur gemeinsamen Stellungnahme der AEM und anderer medizinischer Fachgesellschaften vom 25.03.2020. https://www.aem-online.de/fileadmin/user_upload/Ralf-Stoecker-Ethischer-Hintergrundkommentar-zur-Stellungnahme-der-Fachverbaende-und-AEM-1.pdf. Zugegriffen: 15. März 2022

[CR13] ZEKO (Zentrale Ethikkommission bei der Bundesärztekammer) (2007). Stellungnahme der Zentralen Kommission zur Wahrung ethischer Grundsätze in der Medizin und ihren Grenzgebieten (Zentrale Ethikkommission) bei der Bundesärztekammer zur Priorisierung medizinischer Leistungen im System der Gesetzlichen Krankenversicherung (GKV) – Zusammenfassung. Dtsch Arztebl.

